# Systemic immune‐inflammation index predicts short‐term mortality in acute ischemic stroke with severe stenosis of internal carotid artery associated pneumonia

**DOI:** 10.1002/brb3.70047

**Published:** 2024-09-30

**Authors:** Yi Yang, Peng He, Yongbo Zhang

**Affiliations:** ^1^ Department of Neurology, Beijing Friendship Hospital Capital Medical University Beijing China; ^2^ Bureau of Frontier Sciences and Basic Research Chinese Academy of Sciences Beijing China

**Keywords:** acute ischemic stroke, internal carotid artery, pneumonia, SII index

## Abstract

**Background:**

We aimed to investigate the relationship between systemic immune‐inflammation index (SII) and short‐term mortality in acute ischemic stroke (AIS) with internal carotid artery (ICA) severe stenosis and stroke associated pneumonia (SAP) patients.

**Methods:**

Information on general demographic, laboratory data, CT angiography, magnetic resonance angiography, or digital subtraction angiography were obtained. The predictive power was evaluated by assessing the area under the receiver operating characteristic (ROC) curve. The logistic regression was performed to assess the association of SII and short‐term mortality in severe stenosis ICA‐AIS and SAP patients.

**Result:**

Among 342 patients with severe stenosis ICA‐AIS and SAP, death occurred in 66 patients during 120 days follow‐up. Multivariate regression analyses indicated that increased SII predicts higher mortality in 120 days follow‐up, and the risk of short‐term mortality in SII > 666.31 × 10^9^/L group is increased 4.671‐fold. Patients with SII > 666.31 × 10^9^/L had higher proportion of male, hypertension, smoking, higher admission NIHSS score, higher systolic blood pressure, and higher proportion of 120 days mortality. Higher SII predicted a worse 120 days mortality was worked out by Kaplan–Meier methods.

**Conclusion:**

An elevated SII was remarkably associated with 120 days mortality in severe stenosis ICA‐AIS and SAP patients.

## Graphical abstract

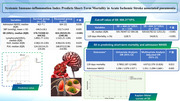

## 1 INTRODUCTION

Atherosclerosis is a major contributor to acute ischemic stroke (AIS), and clinical characters about stroke with internal carotid artery (ICA) stenosis are variable, including asymptomatic, minor stroke, severe disabling stroke, or even death (W. Y. Huang et al., 2018; Nicolaides et al., 2010). There is a study identified that 18.55% of Chinese AIS patients with severe stenosis ICA were dead in 5‐year follow‐up, and stroke‐associated pneumonia (SAP) is one of the main complications in stroke patients (W. Y. Huang et al., 2018). SAP is described as pneumonia that occurs after stroke (Teramoto, 2009). SAP has negative impact on stroke outcomes, such as higher mortality during hospital admission, worse functional outcomes, longer length of hospitalization, and higher hospital costs (Finlayson et al., 2011; Hong et al., 2008; Sellars et al., 2007; Teh et al., 2018).

Immunologic changes and neuroinflammation play key roles in pathogenesis of atherosclerosis. Immune‐inflammation index contributes to AIS, including neutrophil‐to‐lymphocyte ratio (NLR) and platelet‐to‐lymphocyte ratio (PLR) (Esenwa & Elkind, 2016). According to our previous study, systemic immune‐inflammation index (SII) runs on hemorrhagic transformation in AIS caused by large‐artery atherosclerosis (Yang, Han, et al., 2021). Meanwhile, it is no doubt that dysphagia is a well‐known reason for SAP, which leads to severity of stroke, as one of short‐term complications, and there is also some accumulating evidence of an association of SAP and poor outcomes in AIS (Wand et al., 2024). Stroke induces immunology and inflammation depression and enhances SAP susceptibility (Hoffmann et al., 2017).

Recently, the SII has emerged as an independent prognostic indicator of poor outcomes in different diseases (Geraghty et al., 2021; Y. Huang et al., 2021; Wang et al., 2021; Yang, Han, et al., 2021). However, there was no research focusing on the association between SII and short‐term mortality in high‐grade stenosis ICA‐AIS and SAP patients. Therefore, we explored whether SII is closely related to short‐term mortality in high‐grade stenosis ICA‐AIS and SAP patients.

## 2 METHODS

### 2.1 Patients

There were 342 consecutive patients within 3 days of first‐ever diagnosed AIS with high‐grade stenosis (70%–99%) or ipsilateral occlusion of ICA and diagnosis as SAP in hospitalization were enrolled in our retrospective research at Department of Neurology in Beijing Friendship Hospital, Capital Medical University, from January 2020 and June 2023.

Patients were collected by following criteria: (1) diagnosed as AIS based on World Health Organization criteria (Hatano, 1976); (2) diagnosed as SAP, which was identified using the 10th revision of International Classification of Diseases (ICD‐10 codes) J12‐J18 and J69 for complications during stroke hospitalization in the Department of Neurology (Nishimura et al., 2023; Teh et al., 2018). Pneumonia was based on a combination of clinical presentations, radiologic signs, and blood test results, such as a new infiltrate or consolidation lung disease on chest X‐ray or CT, combined with more than one of the following clinical signs: fever, cough, sputum, worsening of respiration, rales, abnormal blood test (high white cell count or C‐reactive protein), positive etiological detection of blood or sputum; (3) laboratory data were collected within 24 h after admission; (4) CT angiography, magnetic resonance angiography, or digital subtraction angiography was performed within 48 h after admission and identified as severe stenosis (70%–99%) or ipsilateral occlusion of ICA by two trained neurologists; ICA classification has the following seven segments: C1, cervical; C2, petrous; C3, lacerum; C4 cavernous; C5, clinoid; C6, ophthalmic; and C7, communicating (Bouthillier et al., 1996). Extracranial ICA, including C1–C4, and intracranial ICA, including C5–C7; (5) 120 days follow‐up by phone and face‐to‐face outpatient.

Patients were excluded if they had (1) had severe stenosis ICA with other causes, such as congenital vascular malformation, arterial dissection, interventional therapy; (2) pulmonary disease; (3) acute or chronic infection, systemic immune diseases, liver diseases, acute kidney disease, hematological diseases, cancer, or were using immunosuppressant drugs; (4) arteritis and autoimmune vasculitis, or moyamoya diseases.

### 2.2 Data collection

Demographic and baseline data of patients were obtained, including age, sex, history of smoking, alcohol consumption, hyperlipidemia, hypertension, diabetes mellitus, prior stroke or transient ischemic attack (TIA), coronary artery disease, chronic kidney disease, atrial fibrillation, admission to the National Institutes of Health Stroke Scale (NIHSS), systolic blood pressure (SBP), ICA imaging examination, and treatment in the hospital. Laboratory parameters were collected the next morning (5:00 a.m.) after admission, including estimated glomerular filtration rate (eGFR), hemoglobin, neutrophil count, lymphocyte count, and platelet count. Each SAP patient was tested for COVID‐19. SII was calculated as the ratio of neutrophil counts to lymphocyte counts multiplied by platelet count (P × N/L). NLR was calculated as the ratio of neutrophil counts to lymphocyte counts (N/L). PLR was calculated as the ratio of platelet counts to lymphocyte counts (P/L).

### 2.3 Follow‐up

Patients were held to a 120 days follow‐up after assessment. The follow‐up was conducted with clinical examination at the first and second week after the first stroke and then every 2 weeks through telehealth communications or face‐to‐face outpatient. Patients’ deaths were clarified, confirmed, and recorded.

### 2.4 Statistical analysis

Normal distribution variables were expressed as mean ± standard deviation (SD), non‐normal distribution variables were expressed as median (interquartile range [IQR]), while the chi‐squared test was used for proportions. The receiver operating characteristic (ROC) curves were applied to test predictors of 120‐day mortality and to determine the substantial cut‐off value. Besides, multiple logistic regression analysis was carried out to analyze the correlation between SII and the incidence of 120‐day mortality in severe stenosis ICA‐AIS and SAP patients. The Kaplan–Meier method was appropriated to draw the survival curves. In the study, variables with *p* <.05 were considered statistically significant, and all statistical analyses were performed using SPSS version 26 (IBM SPSS).

## 3 RESULTS

### 3.1 Baseline characteristics

Of the patients included in the study, there were 342 patients diagnosed with severe stenosis ICA‐AIS‐associated pneumonia enrolled in the current research and follow‐up 120 days after AIS onset in total. The mortality rate in 120 days after stroke onset is 19.30%. The median age was 65.6 ± 10.8 years, and 171 of 342 (50.0%) patients were males. Table 1 shows the baseline demographic characteristics and medical parameters of research participants. Patients in decreased group were more prone to older females and had higher initial NIHSS score and systolic blood pressure. According to laboratory tests, patients in decreased group had lower lymphocyte count (1.89 × 10^9^/L vs. 1.52 × 10^9^/L, *p *= .004), higher NLR (2.58 vs. 4.96, *p *= .029) and higher SII (576.72 × 10^9^/L vs. 824.29 × 10^9^/L, *p* ˂.001) (Table 1).

**TABLE 1:** Baseline characteristics of living status during 120 days in severe stenosis internal carotid artery‐acute ischemic stroke (ICA‐AIS) and stroke associated pneumonia (SAP) patients.

Variables	Survival group (*n* = 276)	Deceased group (*n* = 66)	*p*
Age(years), mean ± SD	65.2 ± 10.2	66.3 ± 11.1	.440
Male, *n* (%)	141 (51.1)	30 (45.5)	.411
Hypertension, *n* (%)	153 (55.4)	42 (63.6)	.227
Diabetes mellitus, *n* (%)	160 (58.0)	45 (68.2)	.128
Hyperlipidemia, *n* (%)	90 (32.6)	24 (36.4)	.561
Prior stroke or TIA, *n* (%)	54 (19.6)	18 (27.3)	.168
Coronary artery disease, *n* (%)	81 (29.3)	21 (31.8)	.694
Chronic kidney disease, *n* (%)	36 (13.0)	12 (18.2)	.280
Atrial fibrillation, *n* (%)	18 (6.5)	6 (9.1)	.463
Smoking, *n* (%)	129 (46.7)	27 (40.9)	.393
Alcohol consumption, *n* (%)	132 (47.8)	24 (36.4)	.093
Admission NIHSS, median (IQR)	4 (2–7)	6 (4–12)	.036
SBP (mmHg), mean ± SD	144.5 ± 18.1	152.7 ± 14.8	.005
**ICA imaging examination, *n* (%)**			
Extracranial ICA severe stenosis	163 (59.0)	41 (62.1)	.678
Left‐side of ICA severe stenosis	126 (45.7)	34 (51.5)	.412
**Treatment in hospital, *n* (%)**			
Intravenous thrombolysis	33 (12.0)	12 (18.2)	.179
Antiplatelet	270 (97.8)	63 (95.5)	.280
Anticoagulation	24 (8.7)	6 (9.1)	.919
Statins	273 (98.9)	63 (95.5)	.055
**Laboratory tests**			
COVID‐19‐associated pneumonia, *n* (%)	46 (16.7)	7 (10.6)	.222
eGFR (mL/min/1.73m^2^), mean ± SD	70.2 ± 25.3	69.5 ± 24.7	.863
Hemoglobin (g/L), median (IQR)	107 (94–116)	105 (90–115)	.276
Neutrophil (10^9^/L), median (IQR)	4.67 (4.20–5.14)	5.72 (4.46–6.97)	.128
Lymphocyte (10^9^/L), median (IQR)	1.89 (1.77–2.01)	1.52 (1.21–1.84)	.004
Platelet (10^9^/L), median (IQR)	219.35 (206.54–232.16)	217.27 (172.31–262.24)	.288
NLR, median (IQR)	2.58 (2.33–2.85)	4.96 (3.01–6.91)	.029
PLR, median (IQR)	124.33 (114.23–134.44)	168.97 (122.98–214.96)	.046
SII (10^9^/L), median (IQR)	576.72 (508.52–644.92)	824.29 (535.93–1240.30)	<.001

Abbreviations: eGFR, estimated glomerular filtration rate; IQR, interquartile range.; NIHSS, National Institutes of Health Stroke Scale; NLR, neutrophil‐to‐lymphocyte ratio; PLR, platelet‐to‐lymphocyte ratio; SBP, systolic blood pressure; SD, standard deviation; SII, systemic immune‐inflammation index; TIA, transient ischemic attack; WBC, white blood cell.

### 3.2 Association of SII with clinical characteristics and poor 120 days outcome

**FIGURE 1:**
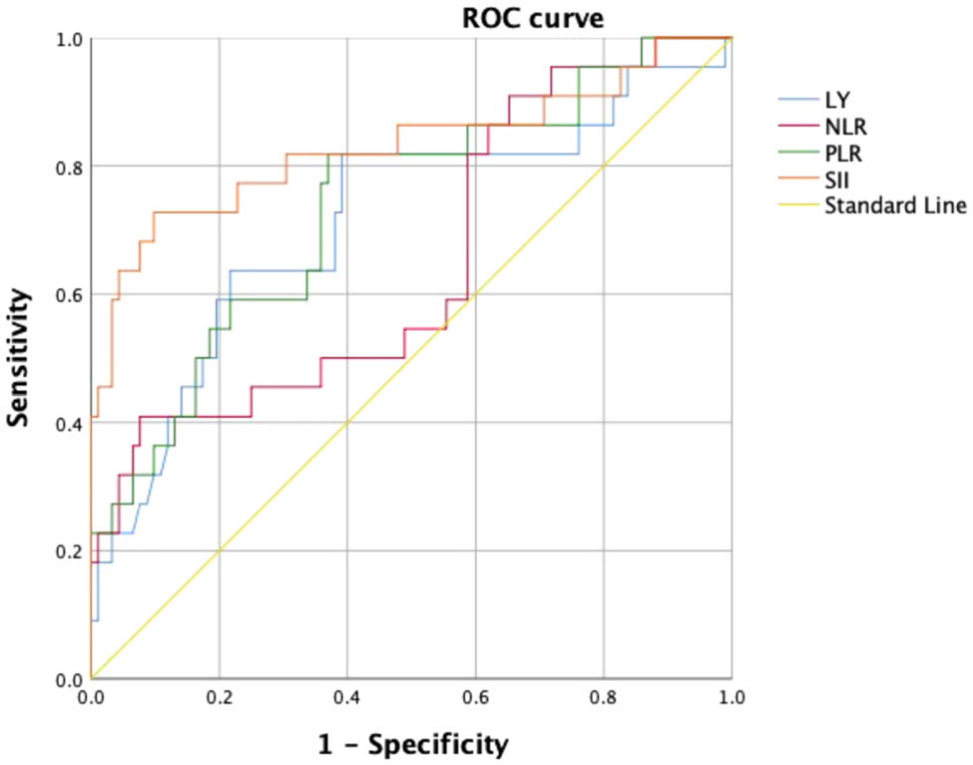
Predictive value of lymphocyte, neutrophil‐to‐lymphocyte ratio (NLR), platelet‐to‐lymphocyte ratio (PLR), and systemic immune‐inflammation index (SII) for living status at 120 days in severe stenosis internal carotid artery‐acute ischemic stroke (ICA‐AIS) associated pneumonia patients. The receive operating characteristic (ROC) curves were used to test predictive power of indicators for 120 days mortality and to determine the optimal cut‐off value. ROC curves demonstrated that the optimal cut‐off value of SII was 666.31 × 10^9^/L (sensitivity 72.7%, specificity 90.2%), the area under the curve (AUC) value 0.830 (0.710–0.949), *p* ˂.001. The AUC value of PLR was 0.736 (0.616–0.856), *p* = .002. The AUC value of lymphocyte was 0.713 (0.581–0.845), *p* = .003. For NLR, the AUC value 0.650 (0.517–0.784), *p* = .027. LY, Lymphocyte.

According to ROC analysis, the area under the curve (AUC) of SII score was 0.830 ([0.710–0.949], *p* ˂.001), indicating SII played a greater role in predicting 120 days overall survival compared to NLR (AUC 0.650 [0.517–0.784], *p *= .027), PLR (AUC 0.736 [0.616–0.856], *p *= .002), and lymphocyte (AUC 0.713 [0.581–0.845], *p *= .003), and the cut‐off value of SII was 666.31 × 10^9^/L with a sensitivity of 72.7% and a specificity of 90.2% (Figure 1).

We performed univariate and multivariate analyses to identify the relationship between SII and 120 days survival status in severe stenosis ICA‐AIS‐associated pneumonia patients. Higher SII was identified to be a major factor of 120 days poor outcome in severe stenosis ICA‐AIS‐associated pneumonia (odds ratio [OR] 1.004 [1.002–1.005], *p* ˂.001; adjusted odds ratio [aOR 1.004] [1.002–1.006], *p* ˂.001) (Table 2).

**TABLE 2:** Univariate and multivariate analysis for the association between indicators and living status at 120 days in severe stenosis internal carotid artery‐acute ischemic stroke (ICA‐AIS) and stroke associated pneumonia (SAP) patients.

	Univariate analysis		Multivariate analysis	
Indictors	OR (95% CI)	*p*	Adjusted OR (95% CI)	*p*
Neutrophil	1.170 (1.081–1.397)	.041	1.115 (0.898–1.384)	.325
Lymphocyte	3.638 (1.329–9.961)	.012	2.045 (0.788–5.307)	.141
NLR	1460 (1.134–1.864)	.002	1.366 (1.059–1.761)	.016
PLR	1.016 (1.007–1.025)	.001	1.011 (1.001–1.021)	.006
SII	1.004 (1.002–1.005)	<.001	1.004 (1.002–1.006)	<.001

*Note*: As continuous variables adjustment for age, sex, diabetes mellitus, prior stroke or TIA, SBP, admission NIHSS, alcohol consumption, statins.

Abbreviations: CI, confidence interval.; NLR, neutrophil‐to‐lymphocyte ratio; OR, odds ratio; PLR, platelet‐to‐lymphocyte ratio; SII, systemic immune‐inflammation index.

To figure out the relationship between SII and 120 days mortality in severe stenosis ICA‐AIS‐associated pneumonia, 342 enrolled patients were divided into two groups based on the cut‐off value of SII (SII ≤ 666.31 × 10^9^/L, *n* = 138; SII > 666.31 × 10^9^/L, *n* = 204). Patients with increased SII tended to have a higher proportion of male (111 [54.4%] vs. 60 [43.5%], *p *= .047), more patients had diabetes mellitus (140 [68.6%] vs. 65 [47.1%], *p* ˂.001), higher proportion of smoking (102 [50.0%] vs. 54 [39.1%], *p *= .0048), higher admission NIHSS score (median: 4 vs. 8, *p*<.001), higher systolic blood pressure (158.7 ± 11.6 mmHg vs. 140.9 ± 20.1 mmHg, *p *= .005), higher SII (×10^9^/L) (920.63 [782.52–1253.21] vs. 505.78 [447.52–584.18], *p* ˂.001), higher proportion of 120 days mortality (54 [26.5%] vs. 12 [8.7%], *p* ˂.001) (Table 3).

**TABLE 3:** Comparisons of baseline characteristics and outcomes between systemic immune‐inflammation index (SII) groups.

Variables	SII ≤ 666.31 × 10^9^/L (*n* = 138)	SII > 666.31 × 10^9^/L (*n* = 204)	*p*
Age (years), mean ± SD	64.6 ± 11.2	67.1 ± 11.4	.360
Male, *n* (%)	60(43.5)	111 (54.4)	.047
Hypertension, *n* (%)	81 (58.7)	114 (55.9)	.606
Hyperlipidemia, *n* (%)	39 (28.3)	75 (36.8)	.102
Diabetes mellitus, *n* (%)	65 (47.1)	140 (68.6)	<.001
Prior stroke or TIA, *n* (%)	24 (17.4)	48 (23.5)	.172
Coronary artery disease, *n* (%)	42 (30.4)	60 (29.4)	.839
Chronic kidney disease, *n* (%)	15(10.9)	33 (16.2)	.166
Atrial fibrillation, *n* (%)	12 (8.7)	12 (5.9)	.318
Smoking, *n* (%)	54 (39.1)	102 (50.0)	.048
Alcohol consumption, *n* (%)	63 (45.7)	93 (45.6)	.991
Admission NIHSS, median (IQR)	4 (2–6)	8 (4–12)	<.001
SBP (mmHg), mean ± SD	140.9 ± 20.1	158.7 ± 11.6	.005
**ICA imaging examination, *n* (%)**			
Extracranial ICA severe stenosis	80 (58.0)	124 (60.8)	.654
Left‐side of ICA severe stenosis	69 (50.0)	91 (44.6)	.377
**Treatment in hospital, *n* (%)**			
Intravenous Thrombolysis	21 (15.2)	24 (11.8)	.354
Antiplatelet	135 (97.8)	198 (97.1)	.664
Anticoagulation	15 (10.9)	15 (7.4)	.259
Statins	135 (97.8)	201 (98.5)	.627
**Laboratory tests**			
COVID‐19‐associated pneumonia, *n* (%)	20 (14.5)	33 (16.2)	.761
eGFR (mL/min/1.73 m^2^), mean ± SD	71.8 ± 26.4	68.0 ± 27.2	.397
Hemoglobin (g/L), median (IQR)	107 (95–118)	105 (88–114)	.128
Neutrophil (10^9^/L), median (IQR)	4.62 (4.14–5.06)	6.02 (4.86–7.21)	.022
Lymphocyte (10^9^/L), median (IQR)	1.90 (1.61–2.17)	1.46 (1.17–1.82)	<.001
Platelet (10^9^/L), median (IQR)	220.35 (190.45–240.46)	216.36 (169.13–220.84)	.076
NLR, median (IQR)	2.01 (1.80–2.79)	5.03 (4.65–8.10)	<.001
PLR, median (IQR)	120.78 (109.23–138.44)	170.97 (128.82–218.87)	<.001
SII, median (IQR)	505.78 (447.52–584.18)	920.63 (782.52–1253.21)	<.001
120 days mortality, *n* (%)	12 (8.7)	54 (26.5)	<.001

Abbreviations: eGFR, estimated glomerular filtration rate; ICA, internal carotid artery; IQR, interquartile range.; NIHSS, National Institutes of Health Stroke Scale; NLR, neutrophil‐to‐lymphocyte ratio; PLR, platelet‐to‐lymphocyte ratio; SBP, systolic blood pressure; SD, standard deviation; TIA, transient ischemic attack; WBC, white blood cell.

**FIGURE 2:**
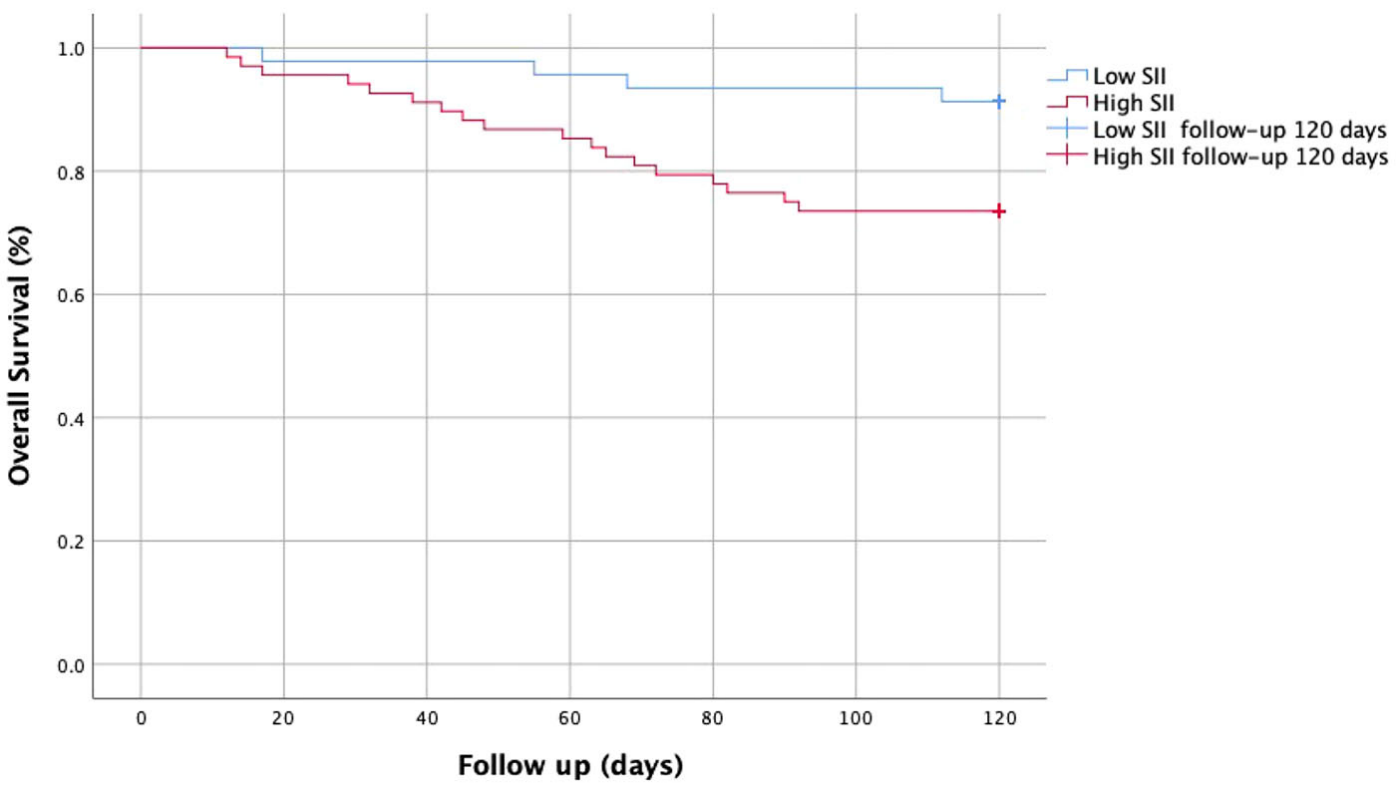
Kaplan–Meier curves of systemic immune‐inflammation index (SII) and groups.

So as to slightly enhance the association between SII and living stature at 120 days in severe stenosis ICA‐AIS‐associated pneumonia patients, multivariate logistic regression was performed. After adjusting for potential confounders, the risk of 120 days mortality in SII > 666.31 × 10^9^/L group is increased 4.671‐fold (*p *= .013), and admission in NIHSS in SII > 666.31 × 10^9^/L group is increased 1351‐fold (*p *= .005) (Table 4). Kaplan–Meier analysis illustrated that a higher SII implied increased 120 days mortality in severe stenosis ICA‐AIS‐associated pneumonia patients (*p* ˂.001) (Figure 2).

**TABLE 4:** Univariate and multivariate analysis for the SII in predicting 120 days mortality and admission National Institutes of Health Stroke Scale (NIHSS) in severe stenosis internal carotid artery‐acute ischemic stroke (ICA‐AIS) and stroke associated pneumonia (SAP) patients.

	Univariate analysis		Multivariate analysis	
Outcomes	OR (95% CI)	*p*	Adjusted OR (95% CI)	*p*
120 days mortality	3.780 (1.187–12.040)	.024	4.671 (1.379–15.826)	.013
Admission NIHSS	1.296 (1.079–1.557)	.006	1.351 (1.094–1.669)	.005

*Note*: As continuous variables adjustment for age, sex, diabetes mellitus, smoking, prior stroke or TIA, SBP, admission NIHSS.

Severe stenosis ICA‐AIS‐associated pneumonia patients’ all‐cause 120 days mortality included fatal stroke (*n* = 22, 33.3%), fatal myocardial infarction and heart failure (*n* = 10, 15.2%), pulmonary complications (*n* = 26, 39.4%), gastrointestinal bleeding (*n* = 7, 10.6%), and kidney failure (*n* = 1, 1.5%).

## 4 DISCUSSION

This is a single‐center retrospective study and demonstrated that higher SII level was positively associated with severe stenosis ICA‐AIS associated pneumonia patients in decreased group. Increased SII was correlated with higher NIHSS score. SII represents an independent predictor of 120 days mortality in severe stenosis ICA‐AIS‐associated pneumonia, which was proved by multivariate logistic regression.

Many studies proved that immune and inflammatory responses play a key role in all stages of vascular lesions formation in atherosclerosis cardiovascular disease (Tsoupras et al., 2018). Atherosclerosis is a major contributor to AIS, and the prevalence of carotid artery stenosis ranges from 4% to 11%, it will be increased by age and race (W. Y. Huang et al., 2018; Rockman et al., 2013). Chronic minor inflammation affected the cerebral vessel wall, which was compromised by atherosclerosis (Dennis & Klaus, 2019). Patients with severe stenosis of ICA usually experience severe neurological deficit, increased incidence of dysphagia, and impaired consciousness, which induce pneumonia (Szabo et al., 2001). In the previous studies, the overall risk of death in 5‐year‐old of patients with high‐grade stenosis of ICA was 17%–21% (Aburahma et al., 2003). The incidence of SAP ranges from 5% to 30% (Ding & Logemann, 2000). There are no studies that have used SII to indicate short‐term outcomes in SAP. In the current study, 120‐day all‐cause mortality in severe stenosis ICA‐AIS associated pneumonia patients was 19.30%. Therefore, severe stenosis ICA‐AIS‐associated pneumonia patients are obviously positively associated with high mortality.

It has been proven that higher SII is correlated with poor outcome in AIS patients treated with intravenous thrombolysis and predicts hemorrhage transformation in AIS (Weng et al., 2021; Yang, Han, et al., 2021). Various inflammatory markers have been regarded as values of AIS, such as neutrophil‐lymphocyte ratio (NLR), platelet‐lymphocyte ratio (PLR) (Sarioglu et al., 2020; Zhang et al., 2020). SII is a novel parameter combined with neutrophil, lymphocyte, and platelet. We are going to discuss the internal mechanism of SII on SAP as follows.

In our study, NLR, PLR, and SII are significantly related to poor outcome in patients with severe stenosis ICA‐AIS‐associated pneumonia through immune and inflammation abnormalities. It also illustrated that the AUC value of SII was superior to NLR and PLR. NLR, as an inflammation marker of carotid plaques stenosis, has been researched in the prediction of stroke prognosis (Köklü et al., 2016; Zhang et al., 2020). In addition, high‐grade PLR level usually predicts inflammatory mediators release and systemic immune dysfunction in response to every step of atherosclerotic plaques erosion (Xu et al., 2019). Meanwhile, PLR plays an important role in early neurological deterioration and unfavorable prognosis in cerebrovascular events (Yang, Xie, et al., 2021). Histopathological studies performed a higher neutrophil count responds to rupture‐prone atherosclerotic plaques on stenosis of ICA (Larinov et al., 2007). Ionita et al. (2010) have demonstrated the role of increased neutrophil count is positive to lipid core size, macrophages and microvessels number, and negative to collagen content and smooth muscle cells, which accelerated the imbalance of inflammatory mechanism. Neutrophil performs as the source of matrix metalloproteinase‐9, which would lead to hemorrhage transformation and symptomatic deterioration (Yamamoto et al., 2012). Neutrophil extracellular traps (NETs) are released by neutrophils and NETs are associated with sterile inflammation in stroke (Alicia et al., 2018). When AIS occurred, platelets were excessively activated and accumulated, which contain a greater amount of fibrinogen and lead to vascular obstruction (Franks et al., 2010). Decreased lymphocytes predict poorer long‐term prognosis in stroke (Kim et al., 2012; Schwartz & Moalem, 2001). Nishinaka et al. (2020) have identified lymphocyte counts were continuing to decrease from 3 weeks before stroke onset to the development of stroke. One of the possible mechanisms is that decreased lymphocyte represents acute stress response; another possible mechanism is that increased pre‐stroke cortisol levels and sympathetic tone by the hypothalamus‐pituitary‐adrenal axis lead to the elevated levels of glucocorticoid and catecholamine hormones, eventually resulting in proinflammatory and aggravating ischemic injury (Park et al., 2011; Xiao et al., 2021). Meanwhile, depression in immunology and inflammation enhanced the susceptibility to SAP and increased mortality (Dirnagl et al., 2007).

The limitations in this study are as follows: First, bias should be considered, on the one hand, this was a single‐center retrospective observation research, and the sample size was relatively small. Our center cannot represent the entire Chinese stroke patients. More prospective and multi‐center studies need to confirm our findings; on the other hand, our retrospective observation research coincides to meet the COVID‐19 epidemic, and some stroke patients may not be admitted to hospital in time. Facing COVID‐19 epidemic, some severe stenosis ICA‐AIS patients moved to postpone interventional therapy, which developed an incidence of complications. Second, we discovered a closed association between SII and 120 days mortality in patients with severe stenosis ICA‐AIS‐associated pneumonia, but further follow‐up is required to confirm the mentioned issues. For example, we should collect the imaging data of infarction area and investigate the association of SII and 120 days mortality in different patterns of infarction area; we should perform follow‐up imaging and laboratory tests to identify the dynamic variable. Further studies should identify the association between baseline and dynamic variables of SII and 120 days mortality in severe stenosis ICA‐AIS‐associated pneumonia patients.

## 5 CONCLUSION

To summarize, we revealed that a higher SII was associated with a greater risk of 120 days mortality in patients with severe stenosis ICA‐AIS‐associated pneumonia, which suggested that SII could dynamically respond to the development of stroke. However, further investigation is needed to monitor the changes of SII on the risk of prognosis in severe stenosis ICA‐AIS‐associated pneumonia in a larger cohort.

## AUTHOR CONTRIBUTIONS

**Yi Yang**: Investigation; conceptualization; writing—original draft; writing—review and editing; validation; methodology; data curation. **He Peng**: Software; formal analysis. **Yongbo Zhang**: Conceptualization; supervision; resources.

## CONFLICT OF INTEREST STATEMENT

The authors declare no conflicts of interest.

## FUNDING INFORMATION

There is no funding relevant to our study.

### PEER REVIEW

The peer review history for this article is available at https://publons.com/publon/10.1002/brb3.70047

## Data Availability

All data generated or analyzed during this study are included in this article. Further enquiries can be directed to the corresponding author.

## References

[brb370047-bib-0001] Aburahma, A. F. , Metz, M. J. , & Robinson, P. A. (2003). Natural history of > or = 60% asymptomatic carotid stenosis in patients with contralateral carotid occlusion. Annals of Surgery, 238, 551–561. 14530726 10.1097/01.sla.0000089856.64262.66 PMC1360113

[brb370047-bib-0002] Alicia, G. C. , Violeta, D. L. , Carolina, P. M. , Ivan, B. , Jesus, M. P. , Jaime, D. G. , Ignacio, L. , & María, A. M. (2018). Myeloid cells as therapeutic targets in neuroinflammation after stroke: Specific roles of neutrophiles ans neutrophil‐platelet interactions. Journal of Cerebral Blood Flow & Metabolism, 38(12), 2150–2164. 30129391 10.1177/0271678X18795789 PMC6282223

[brb370047-bib-0003] Bouthillier, A. , van Loveren, H. R. , & Keller, J. T. (1996). Segments of the internal carotid artery: A new classification. Neurosurgery, 38(3), 432–433. 10.1097/00006123-199603000-00001 8837792

[brb370047-bib-0004] Dennis, W. , & Klaus, L. (2019). Immunity and Inflammation in atherosclerosis. Circulation Research, 124(2), 315–327. 30653442 10.1161/CIRCRESAHA.118.313591 PMC6342482

[brb370047-bib-0005] Ding, R. , & Logemann, J. A. (2000). Pneumonia in stroke patients: A retrospective study. Dysphagia, 15, 51–57. 10.1007/s004550010001 10758186

[brb370047-bib-0006] Dirnagl, U. , Klehmet, J. , Braun, J. S. , Harms, H. , Meisel, C. , Ziemssen, T. , Prass, K. , & Meisel, A. (2007). Stroke‐induced immunodepression: Experimental evidence and clinical relevance. Stroke: A Journal of Cerebral Circulation, 38, 770–773. 10.1161/01.STR.0000251441.89665.bc 17261736

[brb370047-bib-0007] Esenwa, C. C. , & Elkind, M. S. (2016). Inflammatory risk factors, biomarkers and associated therapy in ischemic stroke. Nature Reviews Neurology, 12, 594–604. 10.1038/nrneurol.2016.125 27615422

[brb370047-bib-0008] Finlayson, O. , Kapral, M. , Hall, R. , Asllani, E. , Selchen, D. , Saposnik, G. , & Canadian Stroke Network, & Stroke Outcome Research Canada (SORCan) Working Group . (2011). Risk factors, inpatient care, and outcomes of pneumonia after ischemic stroke. Neurology, 77, 1338–1345. 10.1212/WNL.0b013e31823152b1 21940613

[brb370047-bib-0009] Franks, Z. G. , Campbell, R. A. , Weyrich, A. S. , & Rondina, M. T. (2010). Platelet‐leukocyte interactions link inflammatory and thromboembolic events in ischemic stroke. Annals of the New York Academy of Sciences, 1207, 11–17. 10.1111/j.1749-6632.2010.05733.x 20955420 PMC3245960

[brb370047-bib-0010] Geraghty, J. R. , Lung, T. J. , Hirsch, Y. , Katz, E. A. , Cheng, T. , Saini, N. S. , Pandey, D. K. , & Testai, F. D. (2021). Systemic immune‐inflammation index predicts delayed cerebral vasospasm after aneurysmal subarachnoid hemorrhage. Neurosurgery, 89(6), 1071–1079. 10.1093/neuros/nyab354 34560777 PMC8600162

[brb370047-bib-0011] Hatano, S. (1976). Experience from a multicentre stroke register: A preliminary report. Bulletin of the World Health Organization, 54(5), 541–553. 1088404 PMC2366492

[brb370047-bib-0012] Hoffmann, S. , Harms, H. , Ulm, L. , Nabavi, D. G. , Mackert, B.‐M. , Schmehl, I. , Jungehulsing, G. J. , Montaner, J. , Bustamante, A. , Hermans, M. , Hamilton, F. , Göhler, J. , Malzahn, U. , Malsch, C. , Heuschmann, P. U. , Meisel, C. , & Meisel, A. (2017). Stroke‐induced immunodepression and dysphagia independently predict stroke‐associated pneumonia—The PREDICT study. Journal of Cerebral Blood Flow & Metabolism, 37(12), 3671–3682. 10.1177/0271678X16671964 27733675 PMC5718319

[brb370047-bib-0013] Hong, K.‐S. , Kang, D.‐W. , Koo, J.‐S. , Yu, K.‐H. , Han, M.‐K. , Cho, Y.‐J. , Park, J.‐M. , Bae, H.‐J. , & Lee, B.‐C. (2008). Impact of neurological and medical complications on 3‐month outcomes in acute ischemic stroke. European Journal of Neurology, 15, 1324–1331. 10.1111/j.1468-1331.2008.02310.x 19049549

[brb370047-bib-0014] Huang, W. Y. , Weng, W. C. , Su, F. C. , & Lin, S. W. (2018). Association between stroke severity and 5‐year mortality in ischemic stroke patients with high‐grade stenosis of internal carotid artery. Journal of Stroke and Cerebrovascular Diseases, 27(11), 3365–3372. 10.1016/j.jstrokecerebrovasdis.2018.07.042 30154052

[brb370047-bib-0015] Huang, Y. , Chen, Y.u , Zhu, Y. , Wu, Q. , Yao, C. , Xia, H. , & Li, C. (2021). Postoperative systemic immune‐inflammation index: a superior prognostic factor of endometrial cancer. Frontiers in Surgery, 8, 704235. 10.3389/fsurg.2021.704235 34746222 PMC8568766

[brb370047-bib-0016] Ionita, M. G. , Van Den Borne, P. , Catanzariti, L. M. , Moll, F. L. , De Vries, J.‐P. P. M. , Pasterkamp, G. , Vink, A. , & De Kleijn, D. P. V. (2010). High neutrophil numbers in human carotid atherosclerotic plaques are associated with characteristics of rupture‐prone lesions. Arteriosclerosis, Thrombosis, and Vascular Biology, 30(9), 1842–1848. 10.1161/ATVBAHA.110.209296 20595650

[brb370047-bib-0017] Kim, J. , Song, T.‐J. , Park, J. H. , Lee, H. S. , Nam, C. M. , Nam, H. S. , Kim, Y. D. , & Heo, J. H. (2012). Different prognostic value of white blood cell subtypes in patients with acute cerebral infarction. Atherosclerosis, 222(2), 464–467. 10.1016/j.atherosclerosis.2012.02.042 22460048

[brb370047-bib-0018] Köklü, E. , Yüksel, I. Ö. , Arslan, Ş. , Bayar, N. , Çağırcı, G. , Gencer, E. S. , Alparslan, A. Ş. , Çay, S. , & Kuş, G. (2016). Is elevated neutrophil‐to‐lymphocyte ratio a predictor of stroke in patients with intermediate carotid artery stenosis? Journal of Stroke and Cerebrovascular Diseases, 25(3), 578–584. 10.1016/j.jstrokecerebrovasdis.2015.10.031 26706445

[brb370047-bib-0019] Larionov, S. , Dedeck, O. , Birkenmeier, G. , & Thal, D. R. (2007). Expression of alpha2‐macroglobulin, neutrophil elastase, and interleukin‐1alpha differs in early‐stage and late‐stage atherosclerotic lesions in the arteries of the circle of Willis. Acta Neuropathologica, 113, 33–43. 10.1007/s00401-006-0134-0 16957923

[brb370047-bib-0020] Nicolaides, A. N. , Kakkos, S. K. , Kyriacou, E. , Griffin, M. , Sabetai, M. , Thomas, D. J. , Tegos, T. , Geroulakos, G. , Labropoulos, N. , Doré, C. J. , Morris, T. P. , Naylor, R. , & Abbott, A. L. (2010). Asymptomatic internal carotid artery stenosis and cerebrovascular risk stratification. Journal of Vascular Surgery, 52. 1486–1496.e5. 10.1016/j.jvs.2010.07.021 21146746

[brb370047-bib-0021] Nishimura, T. , Matsugaki, R. , & Matsuda, S. (2023). Physical rehabilitation and post‐stroke pneumonia: A retrospective observational study using the Japanese diagnosis procedure combination database. Neurology International, 15(4), 1459–1468. 10.3390/neurolint15040094 38132973 PMC10745980

[brb370047-bib-0022] Nishinaka, T. , Yamazaki, Y. , Niwa, A. , Wake, H. , Mori, S. , Yoshino, T. , Nishibori, M. , & Takahashi, H. (2020). Alterations of lymphocyte count and platelet volume precede cerebrovascular lesions in stroke‐prone spontaneously hypertensive rats. Biomarkers, 25(3), 305–313. 10.1080/1354750X.2020.1750703 32285702

[brb370047-bib-0023] Park, B.‐J. , Shim, J.‐Y. , Lee, H.‐R. , Lee, J.‐H. , Jung, D.‐H. , Kim, H.‐B. , Na, H.‐Y. , & Lee, Y.‐J. (2011). Relationship of neutrophil‐lymphocyte ratio with arterial stiffness and coronary calcium score. Clinica Chimica Acta, 412(11–12), 925–929. 10.1016/j.cca.2011.01.021 21266168

[brb370047-bib-0024] Rockman, C. B. , Hoang, H. , Guo, Y.u , Maldonado, T. S. , Jacobowitz, G. R. , Talishinskiy, T. , Riles, T. S. , & Berger, J. S. (2013). The prevalence of carotid artery stenosis varies significantly by race. Journal of Vascular Surgery, 57, 327–337. 10.1016/j.jvs.2012.08.118 23177534

[brb370047-bib-0025] Sarioglu, O. , Capar, A. E. , Bas Sokmez, D. F. , Topkaya, P. , & Belet, U. (2020). Relationship between the first pass effect and the platelet‐lymphocyte ratio in acute ischemic stroke. Interventional Neuroradiology, 27(4), 523–530. https://journals.sagepub.com/doi/10.1177/1591019920976251 33236686 10.1177/1591019920976251 PMC8580539

[brb370047-bib-0026] Schwartz, M. , & Moalem, G. (2001). Beneficial immune activity after CNS injury: Prospects for vaccination. Journal of Neuroimmunology, 113(2), 185–192. 10.1016/S0165-5728(00)00447-1 11164901

[brb370047-bib-0027] Sellars, C. , Bowie, L. , Bagg, J. , Sweeney, M. P. , Miller, H. , Tilston, J. , Langhorne, P. , & Stott, D. J. (2007). Risk factors for chest infection in acute stroke. A prospective cohort study. Stroke: A Journal of Cerebral Circulation, 38, 2284–2291. 10.1161/STROKEAHA.106.478156 17569875

[brb370047-bib-0028] Szabo, K. , Kern, R. , Gass, A. , Hirsch, J. , & Hennerici, M. (2001). Acute stroke patterns in patients with internal carotid artery disease: A diffusion‐weighted magnetic resonance imaging study. Stroke: A Journal of Cerebral Circulation, 32, 1323–1329. 10.1161/01.STR.32.6.1323 11387494

[brb370047-bib-0029] Teh, W. H. , Smith, C. J. , Barlas, R. S. , Wood, A. D. , Bettencourt‐Silva, J. H. , Clark, A. B. , Metcalf, A. K. , Bowles, K. M. , Potter, J. F. , & Myint, P. K. (2018). Impact of stroke‐associated pneumonia on mortality, length of hospitalization, and functional outcome. Acta Neurologica Scandinavica, 138(4), 293–300. 10.1111/ane.12956 29749062

[brb370047-bib-0030] Teramoto, S. (2009). Novel preventive and therapeutic strategy for post‐stroke pneumonia. Expert Review of Neurotherapeutics, 9, 1187–1200. 10.1586/ern.09.72 19673607

[brb370047-bib-0031] Tsoupras, A. , Lordan, R. , & Zabetakis, I. (2018). I nflammation, not cholesterol, is a cause of chronic disease. Nutrients, 10, 604. 10.3390/nu10050604 29757226 PMC5986484

[brb370047-bib-0032] Wand, R. H. , Lu, A. L. , Li, H. P. , Ma, Z. H. , Wu, S. B. , Lu, H. J. , Wen , W. Z. , Huang , Y. , Wang , L. X. , & Yuan , F. (2024). Prevalence, predictors, and outcomes of acute respiratory distress syndrome in severe stroke. Neurological Sciences, 45(6), 2719–2728. https://link.springer.com/article/10.1007/s10072‐023‐07269‐8 38150131 10.1007/s10072-023-07269-8

[brb370047-bib-0033] Wang, J. , Zhou, D. , Dai, Z. , & Li, X. (2021). A ssociation between systemic immune‐inflammation index and diabetic depression. Clinical Interventions in Aging, 16, 97–105. 10.2147/CIA.S285000 33469277 PMC7810592

[brb370047-bib-0034] Weng, Y. , Zeng, T. , Huang, H. , Ren, J. , Wang, J. , Yang, C. , Pan, W. , Hu, J. , Sun, F. , Zhou, X. , Qiu, H. , Gao, Y. , Gao, B. , Chi, L. , & Chen, G. (2021). Systemic immune‐inflammation index predicts 3‐month functional outcome in acute ischemic stroke patients treated with intravenous thrombolysis. Clinical Interventions in Aging, 16, 877–886. 10.2147/CIA.S311047 34040364 PMC8143961

[brb370047-bib-0035] Xiao, J. , Qiu, Q.‐W. , Qin, C. , Tao, R. , Qiao, S.‐Y. , Chen, M. , Pan, D.‐J. , & Tian, D.‐S. (2021). Dynamic changes of peripheral blood lymphocyte subsets in acute ischemic stroke and prognostic value. Brain and Behavior, 11(1), e01919. 10.1002/brb3.1919 33111494 PMC7821621

[brb370047-bib-0036] Xu, J.‐H. , He, X.‐W. , Li, Q. , Liu, J.‐R. , Zhuang, M.‐T. , Huang, F.‐F. , & Bao, G.‐S. (2019). Higher platelet‐to‐lymphocyte ratio is associated with worse outcomes after intravenous thrombolysis in acute ischemic stroke. Frontiers in Neurology, 10, 1192. 10.3389/fneur.2019.01192 31798520 PMC6864121

[brb370047-bib-0037] Yamamoto, Y. , Osanai, T. , Nishizaki, F. , Sukekawa, T. , Izumiyama, K. , Sagara, S. , & Okumura, K. (2012). Matrix metalloprotein‐9 activation under cell‐to‐cell interaction between endothelial cells and monocytes: Possible role of hypoxia and tumor necrosis factor‐α. Heart and Vessels, 27(6), 624–633. 10.1007/s00380-011-0214-5 22234512

[brb370047-bib-0038] Yang, Y. , Han, Y. F. , Sun, W. D. , & Zhang, Y. B. (2021). Increased systemic immune‐inflammation index predicts hemorrhagic transformation in anterior circulation acute ischemic stroke due to large‐artery atherosclerotic. International Journal of Neuroscience, 133(6), 629–635. https://www.tandfonline.com/doi/full/10.1080/00207454.2021.1953021 34233123 10.1080/00207454.2021.1953021

[brb370047-bib-0039] Yang, Y. , Xie, D. , & Zhang, Y. B. (2021). Increased platelet‐to‐lymphocyte ratio is an independent predictor of hemorrhagic transformation and in‐hospital mortality among acute ischemic stroke with large‐artery atherosclerosis patients. International Journal of General Medicine, 14, 7545–7555. 10.2147/IJGM.S329398 34754227 PMC8570380

[brb370047-bib-0040] Zhang, W. B. , Zeng, Y. Y. , Wang, F. , Cheng, L. , Tang, W. J. , & Wang, X. Q. (2020). A high neutrophil‐to‐lymphocyte ratio predicts hemorrhagic transformation of large atherosclerotic infarction in patients with acute ischemic stroke. Aging, 12(3), 2428–2439. 10.18632/aging.102752 32028265 PMC7041750

